# A multimodal strategy to improve race/ethnic group equity in administration of neutralizing monoclonal antibody treatment for COVID-19 outpatients

**DOI:** 10.1017/cts.2022.526

**Published:** 2023-02-10

**Authors:** Lindsey E. Fish, Tiffany Bendelow, Sarah Gardiner, Matthew K. Wynia, Bethany M. Kwan, Mika K. Hamer, Adit A. Ginde, Rebecca Hanratty

**Affiliations:** 1 Division of General Internal Medicine, Denver Health and Hospital and University of Colorado School of Medicine, Denver, USA; 2 Department of Medicine, Denver Health and Hospital, Denver, USA; 3 Denver Health and Hospital, Denver, USA; 4 Department of Internal Medicine, University of Colorado School of Medicine, Aurora, USA; 5 Department of Emergency Medicine, University of Colorado School of Medicine, Aurora, USA; 6 Department of Health Systems, Management, and Policy, Colorado School of Public Health, Aurora, USA

**Keywords:** SARS-CoV-2 monoclonal antibodies, health equity, COVID-19, race/ethnic disparity, community-based

## Abstract

**Introduction:**

Racial and ethnic minority groups have higher rates of SARS-CoV-2 infection, severe illness, and death; however, they receive monoclonal antibody (mAb) treatment at lower rates than non-Hispanic White patients. We report data from a systematic approach to improve equitable provision of COVID-19 neutralizing monoclonal antibody treatment.

**Methods:**

Treatment was administered at a community health urgent care clinic affiliated with a safety-net urban hospital. The approach included a stable treatment supply, a same-day test and treat model, a referral process, patient outreach, and financial support. We analyzed the race/ethnicity data descriptively and compared proportions using a chi-square test.

**Results:**

Over 17 months, 2524 patients received treatment. Compared to the demographics of county COVID-19-positive cases, a greater proportion of patients who received mAb treatment were Hispanic (44.7% treatment vs. 36.5% positive cases, *p* < 0.001), a lower proportion were White Non-Hispanic (40.7% treatment vs. 46.3% positive cases, *p* < 0.001), equal proportion were Black (8.2% treatment vs. 7.4% positive cases, *P* = 0.13), and equal proportion occurred for other race patients.

**Discussion:**

Implementation of multiple systematic strategies to administer COVID-19 monoclonal antibodies resulted in an equitable race/ethnic distribution of treatment.

## Introduction

Severe acute respiratory syndrome coronavirus 2 (SARS-CoV-2) was identified in the United States in early 2020 and rapidly spread throughout the country [1–3]. Coronavirus disease 2019 (COVID-19) causes varying clinical presentations from an asymptomatic state to severe illness and death [4–5]. Risk factors for severe illness include older age, diabetes, chronic kidney disease, obesity, and other chronic medical conditions [6–9]. Additionally, Hispanic, Black, Asian, and other non-White patients experience higher rates of infection, severe illness, and death from COVID-19, likely due to factors associated with lower socioeconomic status including crowded housing, use of public transportation, essential jobs that cannot be performed remotely, poorer access to health care, and a higher prevalence of chronic medical conditions [10,11].

In November 2020, the US Food and Drug Administration issued Emergency Use Authorizations (EUA) for two monoclonal antibody (mAb) treatments for high-risk patients with COVID-19 [12,13]. Since then, additional mAb agents have been authorized. These ambulatory therapeutics, delivered intravenously or subcutaneously, can substantially reduce the risk of severe illness, hospitalization, and death from COVID-19 [14–18]. Available and utilized agents have varied over time, in response to the evolution of the SARS-CoV-2 virus and the emerging variants. The EUAs authorize treatment of non-hospitalized patients, with mild-moderate COVID-19 infection, ages 12 and older, weighing 40 kg or more. In addition, qualifying patients need to have conditions that would put them at higher risk for hospitalization or death including older age, chronic medical conditions, or minority race or ethnicity [19].

Unfortunately, despite their higher disease burden and higher rates of having conditions that would qualify them for mAb therapy, Hispanic (any race), Black, Asian, and other non-white patients have received monoclonal antibody treatments at substantially lower rates than White Non-Hispanic patients [20–22]. Specifically, Hispanic patients receive mAb 58% less often than non-Hispanic patients, Black patients receive mAb 22% less often than White patients, Asian patients receive mAb 48% less often than White patients, and Other race patients receive mAb 47% less often than White patients when analyzing national data from November 2020 to August 2021 [20]. Real-world evidence supports the effectiveness of mAb treatment in these racial/ethnic minority groups at preventing hospitalization and death [22–24]. In other diseases, these groups have experienced barriers to care including access to health care, limited or no health insurance, language barriers, and low health literacy at rates that are higher than White patients [25–27]. Specific to COVID-19 treatment, Hispanic and Black patients are less likely to receive mAb treatment due to insurance status, language, and other social factors [28]. Additionally, Hispanic, non-White, and non-English speaking patients are more likely to decline mAb treatment [29]. The location of treatment administration also impacts racial/ethnic minority group access [30].

To address the disproportionate incidence of COVID-19 infection, hospitalizations, and death in racial/ethnic minority groups in Denver, CO, we designed a multimodal mAb delivery strategy to minimize racial/ethnic disparities in access to this outpatient treatment [31]. This strategy addressed issues of location of mAb treatment, limitations of treatment supply, patient education/messaging, language barriers, and inadequate insurance coverage. Here we report the outcomes of this strategy on the racial/ethnic distribution of mAb treatment at one outpatient community health urgent care clinic associated with a safety-net hospital system in metro Denver, CO.

## Methods

### Design

This is a retrospective case review of an urgent care clinic-based model for delivery of mAb treatment for COVID-19. We used administrative data to evaluate racial and ethnic characteristics of patients accessing care relative to county demographics of those diagnosed with COVID-19.

### Outcome

The outcome of this study is the rate of mAb treatment administration for SARS-CoV-2 infection by patient race and ethnicity following a multi-component systematic approach designed to improve treatment uptake.

### Setting and Population

Denver Health and Hospital Authority (DHHA) is an urban safety-net health system, serving one-third of the population of Denver County, comprising a safety-net hospital, ten federally qualified community health centers, and 18 school-based clinics. The DHHA Federico F. Peña Southwest Urgent Care Clinic (PUCC) is a Federally Qualified Health Center (FQHC) located at the intersection of four Denver neighborhoods that have high concentrations of medically underserved populations including 20% non-English-speaking adults, 70% identifying as Hispanic, 51% low-income households, and 20% living below the poverty level [32]. In part due to its affiliation with DHHA, the clinic also draws patients from across the Denver metro area, therefore serving a racially, ethnically, and economically diverse population. Integration within an FQHC community health clinic allows this urgent care clinic to provide treatment to patients regardless of age, language, insurance status, or ability to pay. On average, the urgent care clinic sees approximately 25,000 visits annually.

### Urgent Care Clinic Delivery Model for mAb Treatment

Our urgent care clinic-based mAb delivery model is characterized by a) sustainable use of personnel, space, and materials, including a stable supply of mAb; b) efficient and safe systems and workflows; and c) flexible and equitable policies for treatment access that prioritized those at highest social and medical risk [33].

### Sustainable Use of Personnel, Space, and Materials

PUCC, as an urgent care clinic, had many of the necessary resources in place to administer mAb, specifically, registered nurses, refrigerated medication storage space, intravenous access ability and supplies, and medication pumps. DHHA worked in collaboration with the state public health department to establish and secure a supply of mAb. Due to a purchase by the federal government, the mAb medication itself was free. The specific mAb delivered varied depending on which agent was authorized and effective based on SARS-CoV-2 variant prevalence. When patient volumes were very high, a conference room was converted to an infusion site and additional nurses from within the organization and supporting organizations were utilized to facilitate the administration of mAb.

### Efficient and Safe Systems and Workflows

**Fig. 1.**
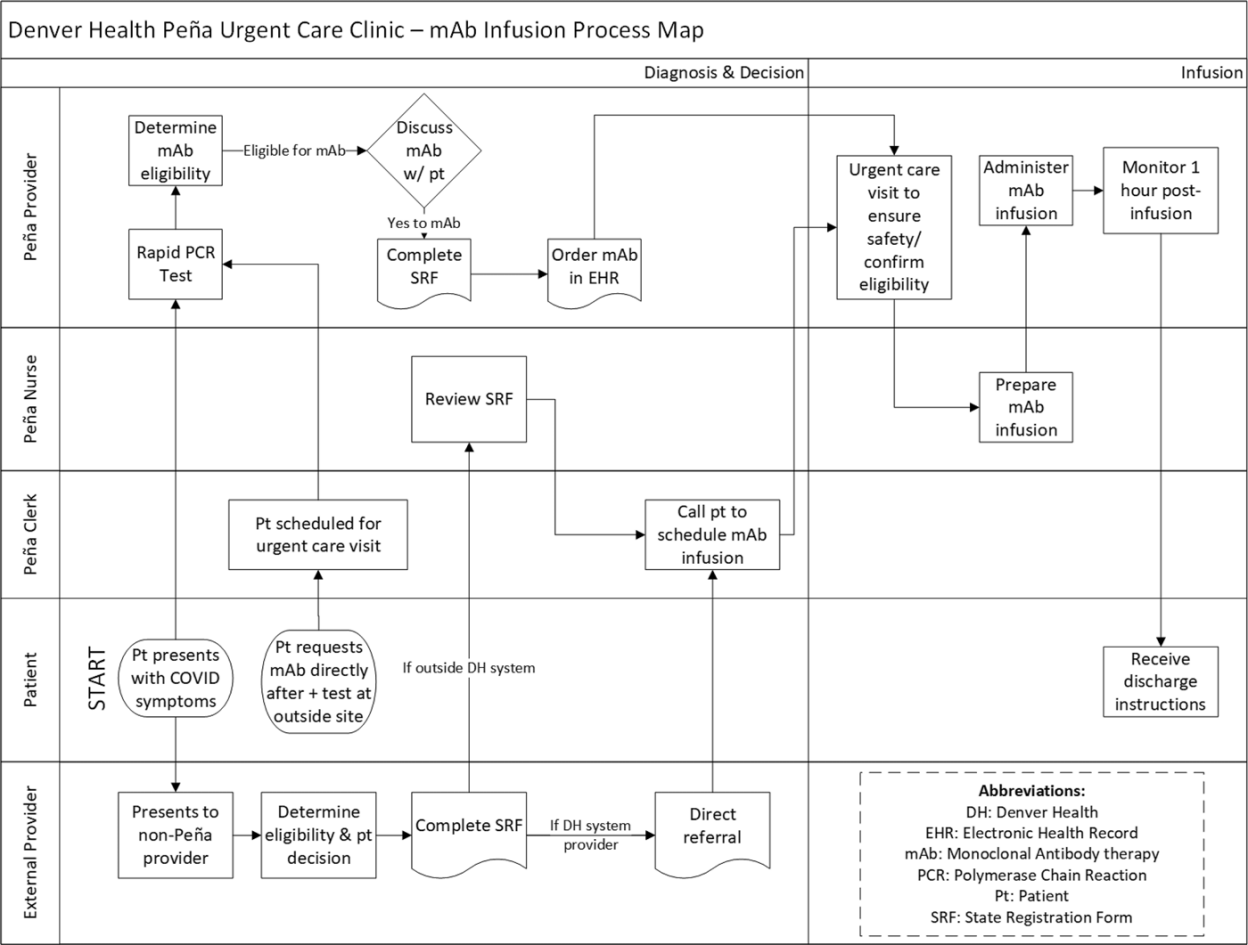
Peña urgent care clinic patient referral and patient flow process map for mAb treatment.

A clinic flow process was created to ensure efficiency and safety when caring for these COVID-19-positive patients (Fig. 1), and staff were trained on the administration of mAbs. Treatment was initially offered in December 2020 to patients via three different processes. The first and most prevalent was from symptomatic patients who presented to the PUCC itself. During that visit, a rapid SARS-CoV-2 PCR test was performed, and if positive and the patient qualified per the EUA, the patient was immediately offered treatment in the same-day visit. Additionally, a process was created for DHHA internal provider-initiated referrals for patients who were tested at other clinical sites within the organization. These referrals were contacted and scheduled for treatment within 1–2 days of the referral being placed. Lastly, external provider-initiated referrals were accepted through a statewide portal system which allowed for scheduling within 1–2 days of the referral being placed.

### Flexible and Equitable Access Policies for Treatment Access

In June 2021, we expanded access by developing a process for proactive patient outreach to offer mAb treatment, thus alleviating the need for patients to rely on a referral from a provider (a barrier due to provider education) or a primary care provider (a barrier to access for patients without a regular source of care). This process was approved through the Colorado Multiple Institutional Review Board. Our electronic health record (Epic Systems, Madison, WI) was used to generate a daily report of all DHHA patients who tested positive for COVID-19 across the system in the previous 24 hours and met treatment criteria per the EUA. Trained staff reviewed these medical records to confirm eligibility by determining date of symptom onset and confirming a high-risk condition. Direct patient outreach was performed on these patients. Funding for this patient outreach staff member was provided by a grant.

In July 2021, we continued to expand access by partnering with the county public health department in Denver, CO. We provided education to public health outreach staff who were contacting COVID-19-positive patients in the community. These outreach workers were taught about the COVID-19 mAb treatment options and who met treatment criteria. When the outreach workers spoke with patients, they gave a brief introduction to the treatment and asked the patient if they would be interested in being contacted by the clinic to learn more about this treatment. If the patient was interested, this generated another daily list of potential patients for treatment.

Utilizing both the DHHA electronic health record list and the public health list, staff contacted patients via telephone, utilizing a certified medical interpreter if needed. The patient was offered mAb treatment and provided education on the treatment itself, the administration process, the risks, and the benefits. Additionally, financial support was offered directly to patients to decrease cost as a barrier. If the patient accepted, they were immediately scheduled and received mAb treatment within two days. DHHA was able to bill commercial insurance, Medicaid, and Medicare patients for the administration costs associated with this treatment. For uninsured patients or those on a fee-based sliding scale program, reimbursement was sought through the federal government’s COVID-19 Uninsured Fund.

*Statistical analysis*: We analyzed the race and ethnicity data descriptively and compared proportions using a chi-square test. We specifically compared the self-reported race/ethnicity of the PUCC treatment group to those who tested positive for COVID-19 in Denver County (self-report) and to the racial/ethnic composition of the Denver County population (census data). Due to the structure of the data, for this analysis race/ethnic groups are mutually exclusive. Hispanic persons could be of any race, and all other races were non-Hispanic. The other/unknown category included persons who identified as more than one race, who did not identify as any of selected race/ethnic group options, or who declined to answer.

## Results

From December 1, 2020, through April 30, 2022, 2524 unique patients received COVID-19 mAb treatment in the PUCC for a total of 2540 doses administered. Prior to June 2021, only 162 patients received treatment and 2365 patients received treatment after June 2021. Three patients received treatment during both timepoints. Of these unique patients, 1129 (44.7%) were Hispanic (any race), 1028 (40.7%) White, 207 (8.2%) Black, 62 (2.5%) Asian, 14 (0.6%) American Indian/Alaska Native, and 6 (0.1%) Native Hawaiian/Pacific Islander (Table 1). There was not a difference between the race/ethnicity of groups treated prior to and after June 2021 (*p* = 0.0807).

**Table 1.** Racial and ethnic distribution of patients receiving mAb treatment at Denver Health and Hospital Peña Urgent Care Clinic compared to the Denver County COVID-19-positive cases

Race/Ethnicity	Denver County COVID-19 Positive Cases [34] (*n* = 128,553)	mAb Treatment Population (*n* = 2524)	Difference (95% CI; *p* value)
Hispanic (any race)	46,881 (36.5%)	1129 (44.7%)	8.23% (6.28, 10.19; *p* < 0.001)
White	59,553 (46.3%)	1028 (40.7%)	−5.57% (3.62, 7.49; *p* < 0.001)
Black	9539 (7.4%)	207 (8.2%)	0.8% (−0.22, 1.94; *p* = 0.13)
Asian	2745 (2.1%)	62 (2.5%)	0.36% (−0.18, 1.05; *p* = 0.21)
American Indian/Alaska Native	924 (0.7%)	14 (0.6%)	−0.15% (−0.23, 0.38; *p* = 0.37)
Native Hawaiian/Pacific Islander	382 (0.3%)	6 (0.1%)	−0.18% (−0.05, 0.27; *p* = 0.10)
Other/Unknown	8526 (6.6%)	78 (2.2%)	−4.44% (3.78, 4.96; *p* < 0.001)

Abbreviations: mAb – monoclonal antibody, *n* – number, CI – Confidence Interval.

Compared to the Denver County COVID-19-positive case population, a greater proportion of Hispanic (44.7% vs. 36.5%, *p* < 0.001) patients received mAb treatment, an equal proportion of Black (8.2% vs. 7.4%, *p* = 0.13), Asian (2.5% vs. 2.1%, *p* = 0.21), American Indian/Alaska Native (0.6% vs. 0.7%, *p* = 0.37), and Native Hawaiian/Pacific Islander (0.1% vs. 0.3%, *p* = 0.10) patients received mAb treatment and a lower proportion of White (40.7% vs. 46.3%, *p* < 0.001) patients received mAb treatment (Table 1). Compared to the Denver County population, a greater proportion of Hispanic (44.7% vs. 27.9%, *p* < 0.001) patients received mAb treatment, an equal proportion of Black (8.2% vs. 8.5%, *p* = 0.59), American Indian/Alaska Native (0.6% vs. 0.5%, *p* = 0.83), and Native Hawaiian/Pacific Islander (0.1% vs. 0.2%, *p* = 0.42) patients received mAb treatment and a lower proportion of White (40.7% vs. 54.3%, *p* < 0.001) and Asian (2.5% vs. 3.8%, *p* = 0.001) patients received mAb treatment (Table 2).

**Table 2.** Racial and ethnic distribution of patients receiving mAb treatment at Denver Health and Hospital Peña Urgent Care Clinic Compared to the Denver County Population

Race/Ethnicity	Denver County Population [35] (*n* = 715,522)	mAb Treatment Population (*n* = 2524)	Difference (95% CI; *p* value)
Hispanic (any race)	199,460 (27.9%)	1129 (44.7%)	16.83% (14.9, 18.8; *p* < 0.001)
White	388,764 (54.3%)	1028 (40.7%)	−13.57% (11.6, 15.5; *p* < 0.001)
Black	61,098 (8.5%)	207 (8.2%)	−0.3% (−0.84, 1.31; *p* = 0.59)
Asian	27,198 (3.8%)	62 (2.5%)	−1.34% (0.66, 1.88; *p* = 0.0004)
American Indian/Alaska Native	3740 (0.5%)	14 (0.6%)	0.03% (−0.19, 0.40; *p* = 0.83)
Native Hawaiian/Pacific Islander	1395 (0.2%)	6 (0.1%)	−0.07% (−0.16, 0.15; *p* = 0.42)
Other/Unknown	33,867 (4.7%)	78 (2.2%)	−2.54% (1.90, 3.04; *p* < 0.001)

Abbreviations: mAb – monoclonal antibody, *n* – number, CI – Confidence Interval.

## Discussion

Implementation of a comprehensive multimodal urgent care clinic-based model for outpatient treatment with monoclonal antibodies against SARS-CoV-2 across a safety-net healthcare system resulted in a more equitable race/ethnic group population-based demographic distribution of treatment recipients. In addition, and potentially more impactful, this model resulted in comparable rates of treatment for racial/ethnic minority groups who were infected with COVID-19 in Denver County. This resulted in a substantial increase in the proportion of Hispanic, Black, Asian, and Other race patients receiving treatment compared to less comprehensive approaches [18–20]. This supports the need for multimodal, complex interventions to address healthcare disparities in the administration of neutralizing monoclonal antibodies for COVID-19 outpatients.

Hispanic, Black, Asian, and other non-White patients experience higher rates of infection, severe illness, and death from COVID-19, likely due to lower socioeconomic status and higher prevalence of chronic medical conditions [10–11], and mAb treatment in these racial/ethnic minority groups is highly effective at preventing hospitalization and death [18–22]. However, multiple studies show that these patients have received monoclonal antibody treatments at substantially lower rates than non-Hispanic White patients. Several identified barriers include insurance status, location of mAb treatment, language, and social vulnerabilities [26–28]. Effective strategies for addressing barriers and improving access to mAb treatment for racial and ethnic minority groups have not been previously published.

This multimodal systematic approach addressed many of the identified and perceived barriers to mAb treatment for racial/ethnic minority groups. First, to address the access to healthcare barrier, we ensured a stable mAb supply with our collaboration with the state public health department. We administered this treatment at a set location in a trusted community-based FQHC urgent care clinic and opened access with same-day and referral-based administration. Second, to address the financial/insurance status barrier, we offered financial support programs to the patients and submitted costs to the COVID-19 Uninsured Relief Fund to minimize the financial impact on the patient. Third, to address the language barrier, all communication was performed in the patient’s native language via a bilingual provider/staff member or with the use of certified interpreter. Fourth, to address the health literacy barrier, we partnered with our safety-net organization and local public health departments to identify potential treatment candidates. Each of these individuals was contacted via direct patient outreach and provided education on the mAb treatment. Patient volume increased significantly after June 2021 when direct patient outreach began. We suspect this contributed to more qualifying patients of all races/ethnicities receiving mAb treatment. This timepoint also coincided with increased availability of mAb, increased provider education and awareness, and increased patient awareness of this treatment due to multiple external factors. Lastly, we utilized a team-based approach, and all staff were familiar with COVID-19 patients, mAb treatments, and caring for underserved populations.

This study is limited to one clinic within a single health system, using some resources that might not be available in other clinical settings. Additionally, there are limitations in the analytic approach which focused only on patient race and ethnicity, unadjusted for other factors that can affect access but for which our data were incomplete, including insurance status, primary language, and neighborhood. We were also unable to assess different patient populations’ reasons for eligibility, as individual-level medical status and high-risk conditions were unavailable.

In conclusion, using a coordinated set of strategies to simultaneously target multiple known barriers to access can dramatically improve receipt of key ambulatory COVID-19 therapeutics for racial and ethnic minority patients. Our experience provides proof of concept that a comprehensive multimodal approach can increase equitable receipt of and decrease racial and ethnic disparities to mAb treatment for COVID-19.
